# Nanocelluloses: Natural-Based Materials for Fiber-Reinforced Cement Composites. A Critical Review

**DOI:** 10.3390/polym11030518

**Published:** 2019-03-19

**Authors:** Ana Balea, Elena Fuente, Angeles Blanco, Carlos Negro

**Affiliations:** Department of Chemical Engineering and Materials, University Complutense of Madrid, Av. Complutense s/n, 28040 Madrid, Spain; anabalea@ucm.es (A.B.); helenafg@ucm.es (E.F.); ablanco@ucm.es (A.B.)

**Keywords:** nanocelluloses, cellulose nanofibers, cellulose nanocrystals, bacterial cellulose, cement, fiber-cement, Hatscheck process

## Abstract

Nanocelluloses (NCs) are bio-based nano-structurated products that open up new solutions for natural material sciences. Although a high number of papers have described their production, properties, and potential applications in multiple industrial sectors, no review to date has focused on their possible use in cementitious composites, which is the aim of this review. It describes how they could be applied in the manufacturing process as a raw material or an additive. NCs improve mechanical properties (internal bonding strength, modulus of elasticity (MOE), and modulus of rupture (MOR)), alter the rheology of the cement paste, and affect the physical properties of cements/cementitious composites. Additionally, the interactions between NCs and the other components of the fiber cement matrix are analyzed. The final result depends on many factors, such as the NC type, the dosage addition mode, the dispersion, the matrix type, and the curing process. However, all of these factors have not been studied in full so far. This review has also identified a number of unexplored areas of great potential for future research in relation to NC applications for fiber-reinforced cement composites, which will include their use as a surface treatment agent, an anionic flocculant, or an additive for wastewater treatment. Although NCs remain expensive, the market perspective is very promising.

## 1. Introduction

The improvement of cement properties through the addition of fibers of different natures (steel, glass, synthetic, and natural) and sizes (macro and microfiber) has been in practice for decades [1,2,3,4,5]. Most researchers have reported that cellulosic fibers are not only non-toxic, renewable, cost-effective, and abundant compared to other fibers (e.g., asbestos, polyvinyl alcohol (PVOH), and polypropylene (PP)), but also provide adequate bonding capacity to cement-based matrices for substantial improvements of toughness, ductility, flexural capacity, and impact resistance [6,7,8]. The drawbacks are long-term durability [9,10,11], mineralization of the fibers [6], and poor dispersion [12]. Therefore, a better scenario would be that in which nano-structurated cellulose materials, also called nanocelluloses (NCs), are used to overcome these problems. Cellulose is the most abundant bio-based polymer on earth with endless applications for engineered materials [13]. NCs have gained substantial consideration due to their exceptional properties that combine both cellulose properties and the unique features of nanomaterials [14]. NCs present several advantages versus cellulose, which include a high surface area, excellent stiffness, unique barrier and optical properties, a light weight, high strength, and the powerful interaction of these products with surrounding species, such as water and inorganic and polymeric compounds [15,16,17,18,19,20,21,22]. Moreover, their inherent cellulose properties, such as biodegradability, renewability, and sustainability, have attracted great interest in many scientific and technical areas as potential sustainable natural-based materials with hundreds of possibilities in multiple industrial sectors [15].

In general terms, NCs can be produced in two different ways: the first is the top-down process, which includes cellulose nanofibers (CNFs) and cellulose nanocrystals (CNCs) production; the second is the bottom-up process, which considers the synthetization of bacterial cellulose (BC). The top-down process to reduce mechanically the size of the fibers down to the nano-scale [23,24,25,26] or to hydrolytically extract and isolate CNCs [27,28,29,30] has been widely described in the past. On the other hand, BC is produced by the fermentation of low-molecular-weight sugar using cellulose-producing bacteria, such as *Komagataeibacter xylinus* species [31].

This diversity of treatments, together with the huge variety of cellulose sources used as a raw material [24,32,33] (e.g., hardwood, softwood, lignocellulosic wastes, cellulose from a biorefinery, seed fibers, bast fibers, grasses, marine animals, such as a tunicate, algae, fungi, and invertebrates) and taking into account the possibility of chemical modification of an NC during or after its production [19,34], lead to a vast array of NCs with different morphologies as well as different physical and chemical properties.

NCs have wide potential in both high- and low-volume industrial applications [35,36,37,38]. The industrial sectors that have the largest potential volume of NCs are paper, paperboard, packaging products, plastics, building and construction materials, textiles, automobile parts, and environmental treatments [22,39,40,41,42,43,44,45,46]. Low-volume applications include medical implants, tissue engineering, drug carriers, wound dressings, aerospace materials, cosmetics, hygiene products, pharmaceuticals, food additives/stabilizers, and paint additives [47,48,49]. In recent years, many research groups and industries have been extensively working on groundbreaking innovations to expand the market for NC products and to open up new potential applications in different areas, such as three-dimensional (3D) printing [50], energy devices [51], printed electronics [52], or energy smart materials [53].

Cementitious composites, such as fiber cement composites (FCCs), have a complex structure that ranges from macro to nanoscale. The incorporation of nanoparticles to increase the contact surface area and its reactivity enables the application of the concepts of high-performance and functionally graded materials. In this scenario, NCs make possible the production of more resistant cement composites or composites with special properties, replacing synthetic polymeric or inorganic fibers. The use of NCs can contribute to achieving the goal of minimizing the carbon footprint of an infrastructure’s materials, driving interest in biodegradable, non-petroleum-based, and low-environmental-impact materials [29,54,55,56,57,58,59,60]. The need for further research is recognized in most research programs worldwide, such as in the E.U.’s Horizon Europe, and many organizations have pointed out the importance of meeting future societal needs by promoting the development of new and better products with a negligible environmental footprint [61].

Most of the NC applications as a reinforcement agent are related to paper or polymer matrices [39,45,59,62,63,64,65,66,67], although, during the last 10 years, studies have also focused on the use of NC to improve cementitious composites materials, such as fiber cement.

Nowadays, the significant number of published papers on this topic calls for a critical review. Only two book chapters provide a general overview of the use of NCs [68,69], summarizing the main improvements and findings from a few research papers. The highly scattered and controversial results of the published studies give this critical review great importance at this time and in this moment so as to explain the differences amongst the behaviors observed in the literature for the efficient future application of NCs in FCCs. Additionally, this review opens up unexplored uses of NCs in the cement industry, giving some ideas for future research.

Therefore, this paper reviews the different ways of using NCs in the Hatscheck process as raw materials or additives. It presents the strong and weak points regarding the conventional raw materials, additives, or procedures. A detailed summary is given on both the mechanism explaining the effects of NCs on the composites’ properties (mechanical properties and rheology) and the variables affecting the reinforcing efficiency of NCs in order to shed light on the controversial aspects related to what seem to be contradictory results. Finally, some new potential applications are reviewed, including their use as a surface treatment agent, an anionic flocculant, or an additive for wastewater treatment.

## 2. Nanocelluloses in the Cement and Fiber-Cement Industry

Two main drawbacks restrict the performance of cellulose fiber cement composites (C-FCCs): (1) the maximum weight content of cellulosic fibers that can be incorporated into the composites; and (2) the long-term durability of the composite [70].

In a Hatschek process, with a good dispersion of the fibers in the fiber cement slurry, the maximum fiber dosage is 10 wt.% due to the effect of fibers on workability, porosity, and density. Additionally, although there is an increase in the toughness using higher dosages, the strength and Young modulus are not further improved. A likely feasible alternative for increasing the reinforcement capacity of the fibers without increasing their percentage is to use cellulose at the nanoscale level.

In general, NCs exhibit enhanced flexural properties compared to cellulose fibers, but they show a brittle behavior since the nanofibrils’ capacity for incipient macrocracks bridging is low as a result of their nano size. In addition, the high specific surface area of NCs leads to an excessive fiber–matrix bonding, which involves a better stress transfer from the matrix to the nanofibers, but, on the contrary, it could contribute to the embrittlement of the composite [71]. Therefore, a combination of NC and cellulose fibers is needed to match the good flexural properties provided by the nanofibers (Young modulus and flexural strength) with the high toughness rendered by the cellulose fibers [71]. Despite this, many of the studies do not combine fibers and NCs. Peter et al. [72] combined micro and nanocelluloses and observed a synergic effect.

Table 1 summarizes the published data on the use of different types of NC to reinforce cement and C-FCCs. It is divided into three parts as a function of the kind of NC used: CNF, nano/micro-crystalline cellulose, and BC. A total of 54% of the publications used CNF, and only three of the papers consider the use of BC in cementitious composites. A total of 32% of the papers studied the effects of CNC or microcrystalline cellulose (MCC) on cement and C-FCCs. The use of CNF to improve the mechanical properties of cement mortar has been studied more than the use of CNF on C-FCCs, and there is nothing on the use of CNC on C-FCCs. However, BC has been preferentially used to improve C-FCCs rather than cement mortar. Table 1 also includes an analysis of the improvements in mechanical properties and other relevant effects on rheological or physical properties. When several doses were used, improvements at the optimal dose or, if possible, the interval of improvements were given. It is observed that the results are very heterogeneous even when the same kind of NC is used. This indicates that there are different factors affecting the efficiency of NCs and this table can help to study them, which is one of the aims of this review.

NCs can be used in different parts of the Hatschek process: (a) as a raw material, combined with cellulose fibers as an alternative to refining; (b) as an additive in coating formulations for surface treatment of the fiberboard; and (c) as a flocculant, since NCs can interact with fibers and mineral particles in a similar way to an anionic flocculant in the wet-end of the machine.

It is important to consider that the high reactive surface of NCs allows for interactions with other components of C-FCCs, such as PVOH fibers, SiO_2_, or alkaline ions, modifying the final properties of the product; however, this has yet to be studied.

### 2.1. Use of NC as a Reinforcing Raw Material to Reduce Pulp Refining Requirements

The refining of pulp is required to improve its ability for processing and to enhance the mechanical properties of C-FCCs. Furthermore, pulp beating increases the durability of composites, as proven by Tonoli et al. [94], due to the improved surface contact area after refining, which enhances the adhesion of the sort fibers to the matrix. Refining is carried out through a disc refiner with a relatively narrow gap between rotor and stator. The main effect is the fibrillation of the fiber surface due to the partial breakage of the bonds between the fibrils. A higher specific surface area increases the capacity of the fibers to bond with the cement matrix and among themselves, which improves the mechanical properties. However, disc refining also causes fibers to be cut-off, decreasing their ability to bridge macrocracks.

**Figure 1:**
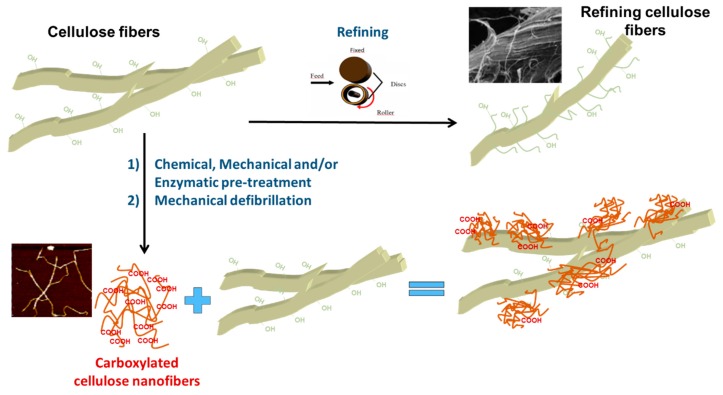
An approach to the potential use of NCs as an alternative to the refining process.

In this scenario, the use of micro/nano celluloses could be an alternative to the refining process (Figure 1).

NC and cellulose fibers have a high tendency to form hydrogen bridges. In the presence of fibers and in the absence of other particles, NCs adsorb on the fiber surface by hydrogen bonding, causing the fibers to be coated with nanofibrils that can have extended tails in the suspension. Coating fibers with NCs increases the specific surface area and the available reactive groups, which can be performed by selecting the appropriate NC [93]. They can be, for example, carboxylic groups if 2,2,6,6-tetramethylpiperidine-1-oxyl (TEMPO)-mediated oxidation is used to prepare NCs. Therefore, interaction between fibers and cement can be improved compared to using refined fibers. The most interesting benefits of using NCs are better fiber–matrix interface adhesion and higher mechanical properties. Table 2 shows the expected effects of using NCs and refining on the fibers and on the C-FCC production process and products.

On the other hand, the refining energy requirement is high and it depends on the desired fibrillation degree, which is usually expressed by the Shopper Riegler scale (°SR) (ISO 5267-1) or Canadian Standard Freeness (mL) (ISO 5267-2). As the fiber length decreases with refining, a compromise between energy consumption and fiber quality has to be achieved for each pulp [95]. Reducing or removing the refining requirements will result in energy savings. However, producing NC is still not cheap enough since it requires high energy and/or chemicals consumption for the low profit margin of the fiber cement product. Moreover, it can be difficult to disperse the NC in the cementitious matrix [86]. These are the main limitations to the use of NC as a raw material in fiber cement. The keys for solving these challenges are: to optimize the dose of NC and to select the right NC.

Improvements in compressive and flexural strength can be achieved by combining the soft refining of fibers with a low dose of NC, leading to a superior-quality product with a higher added value. In this way, refined fibers bridge macrocracks and nanofibers bridge microcracks [71].

The combination of NCs with fibers to replace the refining of fibers has been explored by Mohammadkazemi et al. [93]. They coated bagasse fibers with BC, previously dispersed in water, before using them as reinforcing fibers in the manufacture of C-FCCs. They observed that coating fibers was more efficient in improving mechanical properties than using BC in a powder or gel form directly in the fiber cement slurry. A dose of 3 wt.% of BC, coating half of the bagasse fibers, was enough to increase by more than 50% the modulus of rupture (MOR), absorb energy, favor internal bonding, and increase by almost 40% the modulus of elasticity (MOE). The values of MOR, fracture energy, internal bonding strength and MOE of the C-FCC without BC (28 days curing) were: 4.71 MPa, 0.1 kJ/m^2^, 2.00 MPa, and 5.77 GPa, respectively. When using BC-coated fibers, these values were: 6.51 MPa, 0.17 kJ/m^2^, 2.99 MPa, and 9.71 GPa, respectively. They observed that the BC increases the interaction of fibers with the matrix and encourages hydration reactions at the fiber surface, which increased fiber–matrix bonding. Furthermore, BCs prevent the entrance of alkaline hydration products into the fiber lumen, protecting them from embrittlement and improving the long-term durability. This protection has been demonstrated by SEM micrographs and EDX analysis of elemental compositions on the surface and inside the lumen of fibers. The Ca/Si ratio on the fiber surface was 5.5, half of that in the lumen (10.6), while in the case of C-FCC with BC it was 5.8, four times higher than inside the lumen (1.4). This demonstrates that the hydration products accumulate on the surface of the fibers coated with BC without entering the lumen [92].

Other authors have studied the effect of combining fibers with nanofibers just by replacing some of the fibers by an NC. Claramunt et al. [70] studied the effect of replacing some of the sisal fibers by sisal mechanical micro-nanofibers obtained by high intensity refining, maintaining an 8 wt.% total cellulose dose. The effect of CNF increased with the percentage of replacement of fibrous raw material, reaching a maximal value of MOE (7.7 GPa) when 75% of fibers were replaced by CNF. In this case, the value of MOE increased up to 114% (from 3.6 to 7.7 GPa) and the value of MOR up to 23% (from 11.6 to 14.3 MPa). However, the fracture energy decreased notably (from 1.51 to 0.244 kJ/m^2^), which indicates the effect of embrittlement. The elasticity modulus and fracture energy decayed notably when all of the fibers were replaced by CNF, which was attributed to the low macrocrack bridging capacity of CNF and the lower friction of fracture, both due to their small length. Similar effects were observed by Ardanuy et al. [71] when fiber cement was prepared only with 3.3 wt.% of CNF, instead of fibers, since the MOE and MOR were improved (from 2.4 GPa and 10.3 MPa, for C-FCC, to 4.1 GPa and 14 MPa, respectively, when all of the fibers were replaced by CNF), but the fracture energy decreased by more than 50% (from 759 to 357 kJ/m^2^). The effects on MOE and MOR were lower when the percentage of CNF was 4 wt.% (they reached 3.4 GPa and 10.5 MPa, respectively) and the fracture energy decreased by more than 80% (from 0.3 to 0.05 kJ/m^2^) [73]. However, the replacement of only half of the fibers by CNF resulted in better mechanical properties; the MOE increased by up to 50% (3.9 GPa) and the fracture energy decreased by less than 50% (0.17 kJ/m^2^).

It is well-known that one of the main drawbacks associated with C-FCCs is their limited durability, which is associated with the sensitivity of cellulose fibers to water, carbonation, and strong alkalis, and the generation of incompatible stresses. Loss of adhesion at the fiber–cement interface and increasing micro and macrocracks contribute to strength and durability losses in C-FCCs. However, few studies have highlighted the effect of NCs on cement and concrete durability. In fact, Onuaguluchi and Banthia [96] have stated that future studies should investigate the effects of CNF and CNC on cement durability properties, among other issues. Claramunt et al. [70] compared the durability of cement reinforced with conventional pulps, CNF, or a combination of both by measuring the flexural modulus after 28 days of humidity chamber curing and 20 wet–dry accelerating aging cycles. The results showed that the flexural strength and fracture energy of C-FCC without CNF or with an amount of CNF lower than 4 wt.% decreased due to the fiber debonding and mineralization. However, the use of high content of CNF (4 wt.% or more) increased the flexural strength and maintained the fracture energy after aging. They explained this effect as a consequence of a densification of the interfacial zones combined with strong interactions between CNF and the matrix [70]. CNF do not have a lumen to suffer mineralization, and they have a higher surface area than fibers. Therefore, the replacement of fibers by CNF decreases the contribution of fiber lumen mineralization, which is responsible for embrittlement, and increases the interaction of both phases, which increases the flexural strength and fracture energy. Porosity has been proven to contribute to the lack of durability in wet/dry cycle aging because it allows water to enter into the matrix to dissolve hydration products, mainly calcium hydroxide, which precipitates again by water evaporation during the dry stage of the cycle, causing fiber mineralization. This effect is reduced when using CNFs, since they decrease porosity [74,76,80,81,84,85,86]. SEM images of the C-FCC with 1 wt.% of CNF and 8 wt.% of bamboo pulp show that the nanofiber bridges between the fibers and the matrix remained after 200 aging cycles, which explains the higher fracture toughness after aging (1.3 MPa/m^1/2^) compared with the C-FCC without CNF (1.0 MPa/m^1/2^) [81]. Aging reduced the fracture energy, MOR, and dynamic MOE of both composites, but the values were still higher for the C-FCC with CNF compared to that without (0.382 kJ/m^2^, 19.9 MPa, and 13.4 GPa versus 0.379 kJ/m^2^, 17.8 MPa, and 12.0 GPa, respectively). Banthia et al. [97] observed that the use of a 0.3% of MCC (vol. fraction) on concrete slabs formulation increased their durability, which was evaluated by measuring the curling of the slabs after aging. The presence of MCC led to crack control and curl reduction. Despite the high increase in MOE and MOR that can be reached, the use of CNF is limited by the fracture energy and the cost of CNFs.

### 2.2. Use of NC as an Additive

The use of NC as an additive in fiber cement production has four main aims: (1) to improve mechanical properties, such as bonding strength, MOE, and MOR; (2) to modify the slurry rheology; (3) to reduce the porosity; and (4) to drive interactions with other components of the slurry.

Most of the studies on cementitious materials showed high improvements with NC doses lower than 1 wt.%, and some of them have proved that the effects of NCs can be reverted if they are overdosed. Despite that, the doses of NC used for the studies carried out on C-FCCs are from 1 to 3 wt.% on solids and some improvements have been observed.

#### 2.2.1. Mechanical Improvement

The use of NC as an additive in fiber cement production has four main effects: (1) it improves the bonding strength, MOE, and MOR; (2) it enhances hardening, mainly after 7 days; (3) it reduces the pull out of the fibers and the shrinkage, especially autogenous shrinkage during hardening, reducing the risk of product losses; and (4) it reduces the porosity and thermal expansion.

The reinforcing mechanism is mainly studied for the use of NCs in cement, but it can be extrapolated to fiber cement products. There are five different interconnected mechanisms:

1. The bridging of microcracks;
2. The increase of fiber–matrix interaction;
3. The increase of hardening kinetics near the NC surface;
4. The protection of the fiber lumen from mineralization; and
5. The decrease of autogeneous shrinkage.

Some authors have obtained different effects when using NC or microcellulose in C-FCCs, which indicates the complexity of the reinforcing mechanism. Doses of CNF lower than 1 wt.% associated with 8 wt.% of fibers have been proved to be sufficient to form stress transfer bridges in nano- and microcracks, which result in a higher MOR of the hybrid composites (19.9 MPa) with respect to the C-FCCs (14.8 MPa) and a higher fracture energy (0.422 versus 0.395 kJ/m^2^) [81]. The bridging of microcracks by NC has been observed by means of SEM in cement mortars too, being one of the mechanisms that was initially proposed to explain the notable improvements in compressive and flexural strength [98,99]. The use of 3 wt.% of dispersed BC in gel form increased by up to 58%, 33%, and 30% the MOR, MOE, and internal bonding strength values of C-FCCs with 6 wt.% of bagasse fibers, reaching an MOR value of 9.16 MPa, and an MOE of 6.26 GPa. Lower, but still notable, improvements in mechanical properties were reached when BC was used as a powder, prepared by freeze-drying and milling (an MOR of 8.48 MPa and an MOE of 5.23 GPa) [93]. This indicates that the addition method has a significant influence on the reinforcing mechanism of NCs.

The specific surface area increases significantly (from 50 to 500 m^2^/g) when cellulose fibers are nanofibrilated [64]. The high hydrogen bonding ability of the CNF, due to its high number of hydroxyl groups and specific and reactive surface compared to the fibers, favors both matrix–fiber and fiber–CNF interactions, as demonstrated by da Correia et al. 2018 [81]. Thus, stress transfer bridges are formed at the fiber–matrix interface, which improves the bonding strength, MOE, and MOR and reduces the pull out of the fibers, as proved by Mohammadkazemi et al. [92,93].

**Figure 2:**
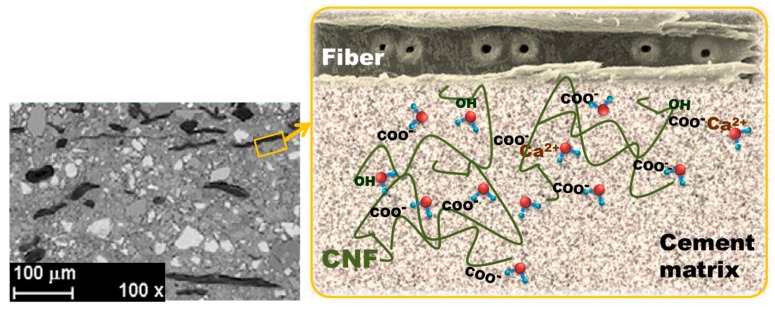
A schematic example of the CNF–water interactions in a cement matrix.

Physical bonding occurs during the hydration of cement when crystals interlock with each other and the fiber surfaces and grow into any other openings and permeable parts of the fiber. Several studies have proved that the hydrophilic and hygroscopic features of CNF can provide a sort of internal water reservoir in the nanofibril network (Figure 2), with a higher concentration of Ca^2+^ ions electrostatically attracted by the anionic nanofibril surface [76,77,78,79,100]. Furthermore, the diffusion of water molecules in the nanofibril network is easier and faster than that in the matrix [84]. These two facts accelerate the production of calcium silicate hydrate (CSH) gel during hydration at the fiber–matrix interface. This causes the accumulation of hydration products at the interface and increases physical bonding between fibers and the matrix, thus improving the mechanical properties and preventing pull out, as proved by means of EDX analysis of elemental compositions in the fiber surface [93] and by X-ray diffraction and FTIR of the composites [76]. The accelerated carbonation curing contributes to the densification of the matrix and increases the dynamic modulus of elasticity [81] and the compressive strength [76].

The effect of nano and microcelluloses on cement hydration has been proved by different researchers. Shuzhen et al. [91] were one of the first to report that the presence of BC accelerated the production of CSH during the hardening of cement. Hoyos el al. [90] studied the cement hydration reaction by thermogravimetric analysis and proved that the use of a 3 wt.% MCC increased the hydration rate during the accelerated curing of the cement paste, as shown by the increase in the intensity of the peaks corresponding to water evaporation and calcium hydroxide (CH) dehydration in a cement paste with 3 wt.% of MCC. Onuaguluchi et al. [79] explained this effect in terms of internal curing. The alkaline matrix hydrolyzes the part of the cellulose that produces organic acids and nonacidic products, and this reaction provides energy to increase the kinetics of the hydration reaction. This was proved by the increase in the hydration heat, proportional to the NC dose, as it had been already observed for fibers by Knill and Kenedy [101] and thoroughly studied by Mezencevova et al. [102]. Mohammadkazemi et al. [93] monitored the temperature during hydration of Portland cement, which was carried out by natural curing of the corresponding C-FCCs and fiber-cement with BNC. They observed that, while the presence of fibers in the C-FCCs decreased the hydration rate and hydration maximum temperature (47 °C) with respect to that for Portland cement (52 °C), the addition of a BC coating on the fibers increased the kinetics of the process with respect to that in the C-FCC and the hydration temperature, which reached 58 °C. Mejdoub et al. [76] proved this by determining the hydration degree for Portland cement with different CNFs based on the amount of the non-evaporable water in the cement paste. The degree of cement hydration at 1, 7, and 28 days increased proportionally to the CNF dose. It was 20%, 40%, and 45%, respectively, for the cement paste and it reached 40%, 49%, and 58%, respectively, for cement containing 0.5 wt.% of CNF. They proposed two additional mechanisms to justify this effect. First, CNF and CNC supply a uniform distribution of cement particles during the hydration process due to the steric stabilization effect. Similar to the mechanism of action of superplasticizers, this steric stabilization increases the hydration process, as was also observed by Cao et al. 2015 [84]. Second, the higher surface area provided by CNF and CNC acts as nuclei to promote the nucleation of hydration product crystals at the early stages of the cement hardening [64]. This favors the accumulation and precipitation of hydrated products in the open pores that were originally filled with water, leading to the formation of a more homogeneous, dense, and compact microstructure than the mixture without CNF addition, as proved by the effect of adding only 0.3 wt.% of CNF to the cement, which reduced the porosity more than the 30% [76]. This contributes to improving the fiber cement’s performance, but it also allows us to control the porosity.

**Figure 3:**
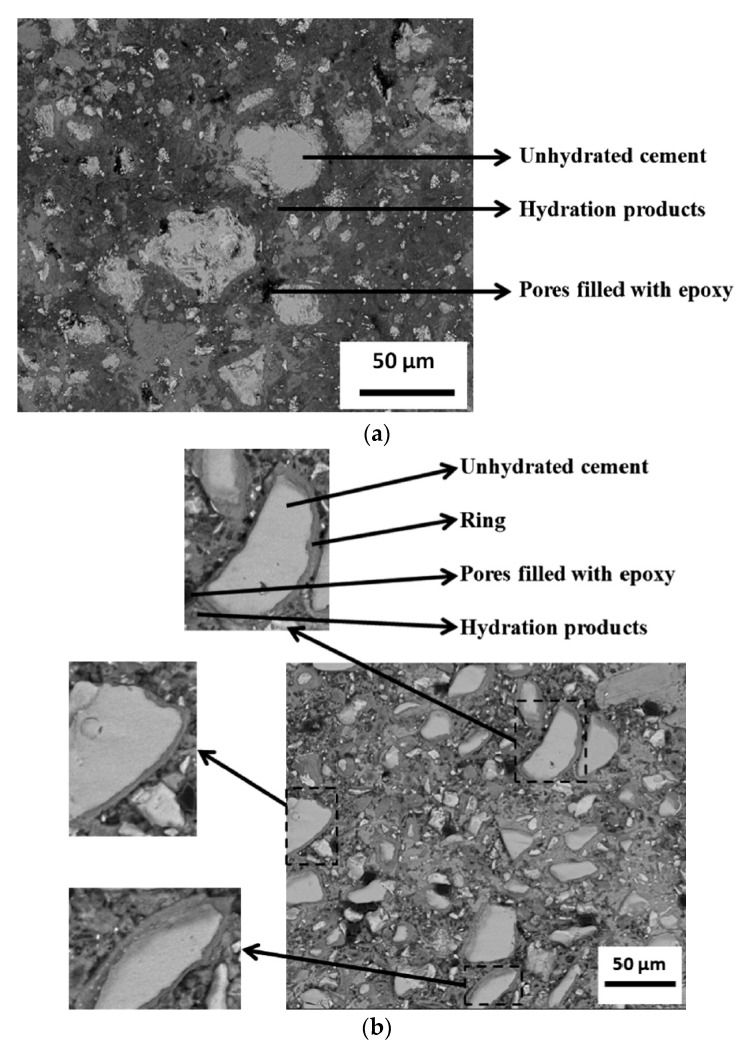
Backscattered scanning electron (BSE-SEM) microscope images of hardened pastes with (**a**) 0% CNC and (**b**) CNC/cement (vol.%) = 1.5 (≈0.77 wt.% CNC) at the age of 7 days. Reprinted from [84] with permission. Copyright Elsevier, 2014.

Cao et al. [84] observed that the concentration of CNC around the unhydrated cement cores that formed a ring or shell, which ultimately lead to the steric stabilization effect, was higher than that in the hydration product (Figure 3). Therefore, the majority of CNCs are adsorbed on the cement surface (>94%) instead of being free in the water phase [85].

The observed reduction in autogenous shrinkage deformations in pastes with CNF has been associated with the effect of CNF on the hydration of cement [68,100]. The accumulation of water in the NC surface, due to swelling, regulates the matrix’s internal moisture and decreases the initial cement hydration rate, but not the global rate. This avoids the destabilization of the matrix by fast water loss as cement hydration proceeds and decreases thermally induced cracking due to the lower heat generation rate. The CNFs retain water and dispatch it to the matrix to partially replenish the emptying cement pores. This has been proved by different studies to be the mechanism that reduces self-desiccation and attenuates early-age deformations [77,78,98]. This is key for composites with a low water/cement ratio, such as the ultra-high-performance concretes. Since internal curing is an effective means to reduce the autogenous shrinkage of high-performance concrete [103], cellulosic fibers are efficient internal curing aids to prevent autogenous shrinkage in concrete [12]. Some researchers have proved that CNFs perform even better [76,77,78,79,100].

#### 2.2.2. Rheology Modifier

The rheology of cement pastes is key for their workability (pumping, spreading, moulding, and compaction). The required rheology depends on the cementitious product; e.g., a C-FCC slurry or an oil well cement must be pumpable, while self-consolidating cement needs to have a very high yield stress.

In freshly prepared cement paste, the small particles interact via colloidal forces, such as Van der Waals, electrostatic repulsion, steric hindrance, and hydrogen bonding forces, and some larger particles interact via direct contact, such as friction or collisions. The presence of NC notably affects these interactions since their large active surface interacts with water, fibers, and particles.

Mohamed et al. [83] studied the effect of CMF on the workability of self-compacting concrete, and proved that the percentage, by mass, of superplasticizer required to obtain a slump of 70 cm and a V-funnel of 10 s was 0.85% of binder for a self-compacting cement with 41% (in volume over volume of cement) CMF, while it was 1.35% in the absence of CMF. However, most studies have shown that the addition of CNF reduces the cement’s workability [72,78,79]. Hisseine et al. [78] proved that the use, in self-compacting concrete, of 0.1 wt.% of CMF was enough to reduce the slump-flow diameter from 785 mm to 538 mm and that a dose of 0.15 wt.% reduced it to 320 mm. In this case, the V-funnel passing time increased from 2.13 to 14.75 s. They also measured the slump-flow diameter of cement with and without CMF and observed that the presence of CMF significantly altered the mixture’s workability, since the slump-flow diameter was reduced from 160 mm to 100, 90, and 85 mm by the use of 0.1, 0.15, and 0.2 wt.% of CMF, respectively. An effect on workability was also confirmed by V-funnel (the passing time increased from 3.5 to 42 s by adding 0.2 wt.% of CMF), and it was consistent with the rheological measurements [78].

Peters et al. [72] showed how the workability of concrete varies nearly linearly in the presence of increasing dosages of CMF and CNF in terms of the water-to-cement ratio and superplasticizer requirements.

The workability of cement depends on its rheological properties. The rheological behavior of fresh cement paste follows a Bingham plastic model. The use of CNF increases the yield stress of the fresh cement, as proved by Mejdoub et al. [76], Hisseine et al. [78], El Bakkari et al. [77], and Nilson and Sargenius [82]. El Bakkari et al. [77] observed that the use of 0.2 wt.% of CNF in a fresh cement paste formulation increased the yield stress from 11.36 to 22 Pa, but the plastic viscosity only increased from 0.5 to 0.68 Pa·s. This effect was due to the high swelling ability of the CNF. Water molecules adhere peripherally to NC, thereby fixing some of the mixing water and thickening the fresh cement paste. The enhancement of the interactions among fibers contributes to an increase in the yield stress of the fiber cement. This contributes to a reduction in the rate of sedimentation of solids particles and the slump, while the limited effect on plastic viscosity still allows us to mix and pump. Furthermore, for certain construction applications, where the fresh paste should retain its shape, a high yield stress is an advantage (e.g., in rigid pavements, stucco, or plastering tiles). Furthermore, pumping is more affected by the plastic viscosity of the cement paste, which increases with the CNF dose too, but with a lower sensitivity to the CNF dose, as proved by the results of El Bakkari et al. [77] and other different studies, which are shown in Table 1 [77,78].

The effect of CNC on rheology depends on the dose [84]. Therefore, CNC can be used to increase the pumping ability of cement or to decrease the slump. This is related to the smallest length of the CNC (100–250 nm) compared to CNF (0.2–3 µm) [14,64]. At low dosages, CNC would tend to adhere to the surface of cement particles rather than agglomerate, carrying water molecules as proved by SEM images [84]. Under shearing, CNC liberates entrapped water molecules and disperses cement particles through electrostatic and steric stabilization while lowering the yield stress [84,87,90]. The yield stress of fresh cement paste decreased from 48.5 Pa to the minimum of 15.9 Pa by using 0.04 wt.% of CNC. As a result, the cement paste flows easily, thus decreasing the energy consumption when pumping. This is desirable for, e.g., reducing superplasticizer demand. At higher CNC loadings, the yield stress increased with the dose, reaching values of 600 Pa for 1.5 wt.% of CNC. For doses higher than 0.2 wt.%, the yield stress increased linearly with the dose due to CNC agglomeration, which requires higher forces to break CNC networks and also the higher reduction of the free water in the matrix [84]. This is interesting for applications where the cement must not have slump, e.g., in 3D printing.

### 2.3. Interaction with Other Components

Different studies on the use of NC in cement mortars and in fiber cement prove that the interaction of NC with the different components of the composite is key for the reinforcing efficiency of NC. For example, the size of the sand used has a great effect on the improvements made by the NC [104]. The use of 3.4 wt.% of CNF on mortar with a cement:silica fume:sand ratio of 0.7:0.3:1 increased the MOE from 5.9 to 6.5 GPa, but decreased the fracture energy from 431 to 78 J/m^2^ when the sand was coarser than the cement particles (the d_50_ of sand was 250 µm and the d_50_ of cement was 15 µm). However, the use of sand with a similar size distribution to cement particles increased the value of MOE and fracture energy in the presence of 3.3% of CNF to 9 GPa and 348 J/m^2^, respectively (the values of MOR and fracture energy of mortar with fine sand and without CNF were 5.6 GPa and 372 J/m^2^, respectively).

**Figure 4:**
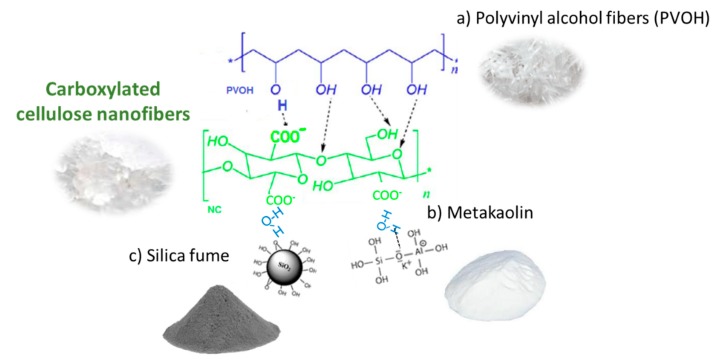
Potential interactions between carboxylated CNF and the different components of the C-FCC matrix, such as (**a**) polyvynil alcohol fibers (PVOH); (**b**) metakaolin; and (**c**) silica fume.

NC can be used to modify raw materials before adding them to the mixture because of the high reactive surface that makes possible the interaction with other components of the C-FCC matrix (Figure 4).

NC can also stablish hydrogen bonding with PVOH fibers to form a composite PVOH/NC with better mechanical properties [105]. There are strong interactions between hydroxyl groups of PVOH and carboxylic groups at the NC surface, which lead to the formation of a rigid network, resulting in improved mechanical properties of the PVOH fibers [106,107]. PVOH fibers could be covered with NC before being added to the mixture and this could increase their interaction with the matrix.

NC can also interact with SiO_2_, i.e., silica fume, which has spherical particles less than 1 μm in diameter (the average is about 0.15 μm) that are very difficult to retain in the matrix [68]. SiO_2_ interacts with NC through the adsorbed water molecules, NC-water-SiO_2_. The amount of silica fume used in fiber cement is small. The dispersion of silica fume in the NC hydrogel could form a suspension of silica fume particles preflocculated with NC or coated with NC depending on the relative doses of silica fume and NC. The high interaction of NC with the cellulosic fibers and matrix would improve the retention of silica fume in fiber cement. The same can be said for metakaolin, although the particle size of metakaolin is smaller than that of cement particles, and it is not as fine as silica fume; thus, it is easier to retain [68,106].

On the other hand, NCs interact with Ca and alkaline ions through electrostatic attractive forces and with anionic flocculants through the attached Ca ions, with some contribution of Van der Waals forces due to the high molecular weight of the polymeric flocculant. Furthermore, NCs can be modified by carboximethylation, cationization, sylilation, grafting with polymers, etc. [14,64]. This allows us to control the interactions with the other components of the composite.

### 2.4. Understanding the Effect of Nanocelluloses

Table 1 shows that the use of NC in cementitious composites has many different effects on mechanical properties, even if the same dose of NC is used. Some researchers showed reductions in the fracture energy or in the compressive strength, while others obtained significant improvements in these properties [74,78].

The mechanical properties of cementitious composites are controlled by at least six main factors: (1) the intrinsic mechanical properties of the reinforcing fibers and/or nanofibers (bending strength, stiffness, tensile strength); (2) the dimensions; (3) the dispersion and orientation of the fibers and/or NCs in the matrix; (4) interactions between reinforcing fibers and the matrix; (5) the effects of NC on cement hydration; and (6) the mechanical properties of the matrix, which are related to the matrix’s composition and microstructure.

These factors depend on several variables that can be optimized: (1) the type of cellulose reinforcement; (2) the cellulose’s or NC’s surface chemistry and morphology, which depend on the mechanical or chemical treatment used for their production; (3) the dose of NC; (4) the way the NCs are incorporated into the composite; (5) the curing process; and (6) the morphology and nature of the components of the matrix, such as sand or silica.

The intrinsic mechanical properties of NCs are similar to those of steel fibers and several others of higher magnitude than those for cellulose fibers. Therefore, this factor is always improved when nanofibers are used in reinforced cement. However, the surface chemistry plays a relevant role in controlling interactions among nanofibrils and the matrix. These interactions increase with the hydrophilic character of the NC’s surface as shown by the 20% shrinkage reduction in a cement reinforced with 0.8 wt.% of TEMPO-oxidized CNF when the amount of carboxylic acid groups was 1.13 mmol COOH/g [77]. Mejdoub et al. [76] observed an increase in cement hardening (up to 66%) when TEMPO-oxidized CNFs were used. The amount of water adsorbed by the nanofibers increases with the carboxylation grade [108], and this improves the water reservoir function of the NC during the cement’s hydration, reducing autogenous shrinkage. Furthermore, the increase in water retention reduces the leaching and bleeding in the cement, as observed by El Bakkari et al. [77].

The kind of NC is a key factor, as proven by studies carried out by Vazquez et al. [87], Hoyos et al. [90], Cengiz et al. [75], and Alshaghel et al. [88]. They used commercial MCC, which has a lower aspect ratio (from 0.04 to 5) and a higher tendency to aggregate in clusters than those of CNF (the aspect ratio can reach values over 100), as showed by SEM images obtained by Alshaghel et al. [88], Cengiz et al. [75], and Tanpichai [109]. The low aspect ratio of MCC and its aggregate-forming clusters decrease its ability to bond cracks, reducing its reinforcing effect. In fact, Cengiz et al. [75] compared the reinforcing effect of commercial NC from Sigma Aldrich, SEM images of which indicate that it could be actually an MCC, with that of CNF produced from waste algae. While the commercial product notably decreased the flexural strength of the cement mortar, the CNF increased it by up to 170%. Most of the researchers working with MCC have observed similar effects on different cement mortars. Anju et al. [89] prepared MCC by limited acid hydrolysis, instead of using commercial MCC, resulting in a product with a larger aspect ratio (from 10 to 80) than the commercial MCC. They observed an increase of 50% of MOR by using 2.5 wt.% of MCC in the cement mortar, but the compressive strength decreased by 21%. The reinforcing effect of MCC was notably improved by means of tetraethyl orthosilicate (TEOS) modification, because of the lower hydrophilic character and the higher siliceous character of the surface of modified MCC. These results prove the effect of morphology and surface nature on NC behavior.

The dispersion of NCs is a key aspect for their efficiency [110]. This has been studied for CNC by Cao et al. [85,86]. They proved that the use of CNCs dispersed in water and sonicated for 2 h increases their effect on cement reinforcement and reduces the large size porosity. They studied the maximum CNC dose that can be used because CNC aggregates retain larger amounts of water and CNC powder can entrap air too.

The curing process must also be taken into account. Hoyos et al. [90] studied the effect of MCC on the standard process of Portland cement hydration (by keeping the specimens for 28 days in a limestone-saturated solution at room temperature) and on accelerated hydration (by keeping the specimens for 7 days in a limestone-saturated solution at 50 °C followed by a dry oven at 60 °C for 48 h). They observed that, while the presence of MCC increased the hydration rate during the accelerated hardening, it decreased the hydration rate when hydration took place at room temperature, as proved by TGA, SEM, and EDX analysis of cured composites with 3 wt.% MCC [90]. The presence of solved polysaccharides in the water resulting from the cellulose hydrolysis could be the responsible for that. Polysaccharides can adsorb on the surface of cement and calcium hydroxide crystals, consuming water molecules in their own hydrolysis reaction, and reducing the availability of water to react with the cement particles, as demonstrated by Pourchez et al. [111,112]. Furthermore, they can complex with calcium ions to reduce the crystallization of CH and calcium silicate hydrate (CSH), as demonstrated by Peschard et al. [113,114]. These phenomena compete with the slow cement hydration process at room temperature. However, when the cement hydration is accelerated by increasing the temperature, the internal curing action of the NC is the predominant effect, resulting in a higher cement hydration grade at the end of the process. This explains the results obtained by Anju et al. [89], since they used natural curing at room temperature. The compressive strength of a cement mortar at 28 days decayed from 42 to 33 MPa and the flexural strength increase was low, from 5 to 5.8 MPa, by using a dose of 2.5 wt.% MCC. The TEOS modification of MCCs protects them from alkaline hydrolysis, which reduces the presence of solved polysaccharides and contributes to an improvement in its reinforcing effect. In this case, the use of TEOS-modified MCCs increased the compressive strength to 65 MPa and the flexural strength to 8 MPa [89].

Amorphous parts of the CNF could suffer hydrolysis too, forming soluble polysaccharides that could affect the hardening depending on cement composition and CNF characteristics. However, the improvements in the hardening rate observed with CNF for natural curing (the degree of hydration after 1 day of curing) increased from 20% to 40% when a dose of 0.5 wt.% CNF was used in the formulation of the cement paste. This proved that the effect of solved polysaccharides is lower than the internal curing effect of the CNF [76].

Not many studies have been carried out with a scan of NC doses in reinforcing cementitious materials. Several studies have been carried out with CNF, and most of them used doses lower than 1 wt.% [75,78,80]. The reinforcing effect of CNF usually increased with a dose up to a maximal value and decreased at high doses because the aggregation of CNF leads to dispersion difficulties and contributes to the formation of weak points, as reported by Mejdoub et al. [76] for CNF prepared by TEMPO-mediated oxidation and by Onuaguluchi et al. [79] for CNF obtained by mechanical fibrillation without pretreatment. In the case of CNC, similar observations were published by Cao et al. [84].

On the other hand, even at the same dose, kind, and morphology of NC, the addition method for NC has a notable influence on its reinforcing effect. This was proved by Mohammadkazemi et al. [93], who prepared a fiber cement composite reinforced with BC. They added lyophilized BC in powder form and water-dispersed BC in gel form. In this case, they tried the direct addition of BC gel to a mixture of cement and fibers and a previous coating of 50% of the fibers with BC by mixing them with a gel containing 0.1 wt.% of dispersed BC overnight before mixing them with the cement. The highest reinforcing effect was obtained in this way because of the additional fiber protective action of the BC adsorbed onto the fibers, which helped to avoid lumen mineralization, and the increase in the hydration rate in the fiber surface. The BC was distributed in the whole matrix when it was added directly to the fiber cement slurry, which reduced the presence of the protective mechanic as a lower amount of BC was adsorbed onto the fibers. The lowest reinforcing effect was obtained by adding the BC in powder form because of the fewer accessible OH groups in the BC powder, resulting in a reduction of the hydrogen bonding ability. The MOE values for C-FCC without BC, with BC-coated fibers, with BC as a powder, and with BC in gel form (the dose of BC was 0.3 wt.% in the three cases) were 5.77, 9.71, 8.48, and 9.16 GPa respectively, which illustrates the relevance of the addition method. The MOR values were 4.71, 6.5, 5.23, and 6.26, respectively.

Claramunt et al. [74] observed that the effect of CNF on fiber cement reinforcement could be magnified by decreasing the particle size of sand. They multiplied by almost six times the reinforcing effect of the CNF and avoided its negative effect on fracture energy by using fine sand instead of coarse sand (the values are given in Section 2.3). Although interactions between the CNF, fibers, and cement were observed, the three components formed a homogeneous paste surrounding coarse sand particles with a low number of interactions with them. However, the use of fine sand particles increased the homogeneity of the matrix and the interaction among sand particles and fibers.

## 3. Further Applications to the C-FCC Production Process

According to studies carried out in other sectors, there are other possible ways to improve the product or the C-FCC production process that have yet to be studied. Here are three possible uses that are worth studying in the future.

### 3.1. Surface Treatment of Fiber Cement Boards

Fiber cement products receive different surface treatments to improve their resistance to water staining, dirt, algae, mold, and extreme climatic conditions and to improve the adhesion of fiber cement corrugated sheets. Most of these treatments consist of coating the surface with a synthetic product, such as acrylic paint or epoxy resins. NC could be used as a coating agent in a promising surface treatment of the composite layers. In this scenario, NCs attach to the surface to improve the adhesion between C-FCC corrugated sheets because NCs have the same nature as the cellulose fibers that are used as a reinforcement agent and enhance the network formation between fibers of different layers via hydrogen bonding.

Additionally, NC can be modified to provide special surface properties in the cement composite, such as hydrophobicity. Modified NC can form a waterproof film when the NC gel is applied, for example by spraying, onto the C-FCC surface. Hydrophobization of TEMPO cellulose nanofibrils has been targeted through coupling amine-functionalized molecules onto the surfaces of NC [115]. This type of NC product may be of interest for fiber cement production.

### 3.2. Use of NC as a Flocculant

The production of C-FCCs by the Hatscheck process requires the use of flocculants to attach the minerals to the cellulosic fibers that form the composite material. This is because of the great difference in nature and density between cellulose and the minerals, leading to poor affinity among them and poor retention [116]. Inorganic particles tend to segregate from the slurry and pass through the wire, without being retained in the sheet, if flocculants are not used. The flocculants induce the aggregation of particles, improving the retention at the primary sheet’s formation and the drainage of water. This makes the formation of the composite sheets feasible and reduces the recirculating load of fine particles in the water system [117].

**Figure 5:**
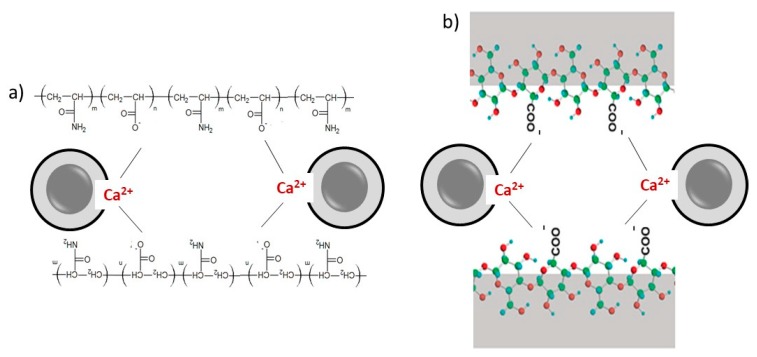
The flocculation interactions between cement grains or hydrates and anionic polyacrylamide (APAM) (**a**) or carboxylated NC as a promising flocculation agent (**b**).

The most common flocculants used in C-FCC production by the Hatscheck process are anionic polyacrylamides (APAMs). This is because the flocculation process is enhanced by the interaction of the Ca^2+^ ions, produced by the cement hydration, with the carboxylic groups of the polymer chains [118] (Figure 5a).

However, the use of APAMs can reduce the flexural strength of the composite. Several studies have been carried out to mitigate the strength deterioration due to the use of APAMs. Negro et al. [119] have shown that this effect is lower when the charge density of APAM increases and the molecular weight decreases. They have also proposed the use of alternative flocculants as dual systems formed by polyethylene oxide and phenolic resins [120,121].

Another approach to improve the mechanical properties of C-FCCs is surface chemical treatment of the fibers to reduce their hydrophility, such as etherification, esterification, silanization, and urethane formation, improving the bonding between cellulose fibers and the cement matrix [122].

Although some advances have been achieved, it is of interest for the industry to find alternative flocculation aids that improve the process’s efficiency without affecting the product quality. In this context, NC could replace APAMs as an alternative flocculant agent with a synergic effect. In this case, the carboxylic groups of NC, produced by TEMPO-mediated oxidation, would attach Ca^2+^ ions, interacting with the cement and other anionic particles by electrostatic forces, as shown in Figure 5b.

Moreover, NCs enhance cement hardening in contrast to the use of APAMs. Although no studies have been published on the use of NCs as flocculant aids in the C-FCCs industry, some researchers have evaluated the flocculation efficiency of NC and cationic NC in other industrial processes [123,124,125,126,127,128,129,130].

Sun et al. [126] reported that anionic CNCs are very effective for the depletion flocculation of colloidal size bacteria, in which the phase separation of bacteria occurs at very low concentrations of CNC (less than 0.1 wt.%). Suopajärvi et al. [127] studied five anionic dicarboxyl acid CNFs with variable charge densities and demonstrated that CNFs with a high charge density (1.7 mmol carboxylic groups/g) and a high nanofibrillation degree produced the best flocculation performance in the coagulation–flocculation treatment of municipal wastewater. One year later, Suopajärvi et al. [128] reported that sulfonated CNF had better flocculation performance than that reported earlier for the dicarboxyl acid CNF due to the sulfonic groups probably having a higher affinity towards iron patches of coagulant than the carboxyl acid groups. Yu et al. [129] demonstrated that CNCs with high carboxylic group content (1.39 mmol carboxylic groups/g) have a remarkable coagulation–flocculation performance in a kaolin suspension with a turbidity removal of 99.5%. Yu et al. [130] reported that CNFs flocculated microalgae via mechanical entrapment and hydrogen bonding.

In the manufacture of C-FCCs, NC may constitute a promising flocculant agent that forms a tridimensional network inside the cement composite in which the anionic charge density of the NC can be easily controlled by different chemical oxidation pretreatments (e.g., by the NaClO concentration used during TEMPO-mediated oxidation pretreatment). Moreover, NC can be grafted with flocculants or chemically modified to obtain the required flocculant efficiency and flocculant properties [131].

### 3.3. Wastewater Treatment

Most modern factories require a closed water circuit for manufacture in order to promote an environmentally friendly process. The accumulation of cations (e.g., K^+^, Na^+^, Ca^2+^) and sulphates (e.g., K_2_SO_4_, Na_2_SO_4_, and 2CaSO_4_·K_2_O_4_) decreases the durability of the fiber cement and affects the process. Therefore, they must be removed from the water before its reuse. Precipitation of alkaline ions is not affordable due to the high solubility of K^+^ and Na^+^. Such methods as osmosis are too expensive and require extensive pretreatment, and ionic exchange resins are also expensive and generate non-biodegradable wastes. Therefore, other methods must be developed. Recent advances in nanoscale science suggest that many of the current water treatment problems might be solved or greatly ameliorated by using NC [42,132]. NCs supported on a biodegradable matrix can be a feasible ionic catcher as they have a very highly reactive surface [133,134]. Additionally, they can be modified to increase their affinity for different compounds (e.g., NC could be useful to remove the excess of flocculant from the wastewater).

**Figure 6:**
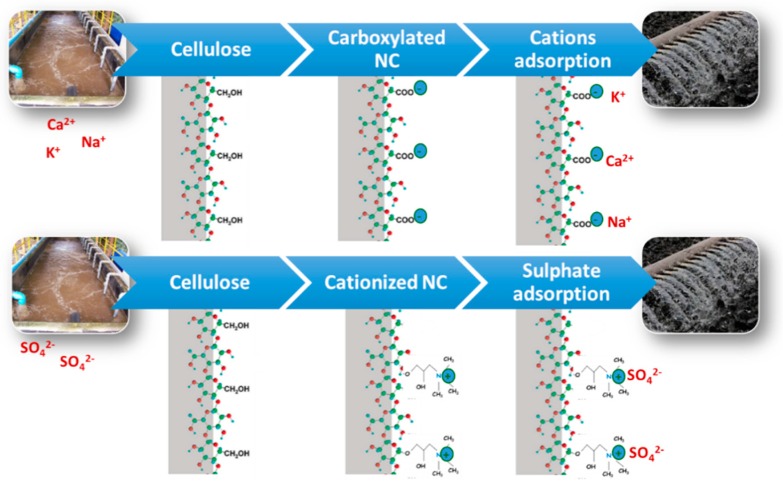
A representation of cation and sulphate adsorption using NC.

Carboxylation of NC is the most-used process, in which the high density of carboxylic acids (COO^−^) could react with cations, such as K^+^, Na^+^ and Ca^2+^, present in the wastewaters and remove them [133]. For removing sulphates, NC should be modified. Cationic NCs are efficient adsorbents to remove negatively charged water ions [135], such as nitrates, phosphates, fluorides, and sulphates (Figure 6). Additionally, cationic CNF displayed a higher selectivity toward multivalent ions (PO_4_^3−^ and SO_4_^2−^) than monovalent ions (F^−^ and NO_3_^−^) [136].

## 4. Market and Sustainability

We have analyzed different promising applications when using NC for functionally graded cement and fiber cement production based on the improvement of the properties of these materials. The major driver for utilizing CNF as a reinforcement for fiber cement products is the possibility of exploiting the high tensile moduli (a single cellulose nanofiber possess a tensile moduli between 100 and 160 GPa). However, the general consensus for the next stage of development is to continue exploring a broader marketplace beyond the conventional “stronger and stiffer” structural reinforcement application, as well as to address sustainable development issues.

### 4.1. Sustainability

An important issue for future market development is to consider the sustainability aspects of using NC. As mentioned above, CNFs are natural, abundant, renewable, bio-degradable, high in strength, and low in weight, making them attractive for developing bio-based, more sustainable building solutions. However, in this aspect, one major question still remains: Are cellulose nanofibrils truly environmentally friendly compared to commercially available engineering materials?

NCs, which are obtained from renewable resources, are an example of a material under development for which a reduced environmental impact is expected compared with existing materials. However, this question is difficult to answer as these products are in their earlier stages and data for functionally graded cement and fiber cement production applications are limited to the research stage only, meaning that just lab-scale data are available.

Life cycle assessment (LCA) has been widely used for eco-design purposes to highlight the bottlenecks and hotspots in the production process and the new material compared with competing products. A few LCA studies have been published that take into account the environmental impacts of NC [137,138,139,140]. These LCAs used a combination of lab- and pilot-scale measurements to look at different processing routes and provide important insights into future large-scale industrial production in the absence of industry data.

These studies show that the main environmental impacts are related to the high energy consumption of fibrillation for all analyzed fabrication route scenarios [140,141]. During the last few years, different pretreatments, prior to fibrillation, have been developed with the aim of reducing the energy consumption of such processes as enzymatic hydrolysis, carboxymetlylation, mechanical refining, and TEMPO-mediated oxidation, and have led to a reduction in the energy requirements for fibrillation from values higher than 100 to only 2–4 Kwh/Kg [14,64].

### 4.2. Market

The commercialization of CNF is still at an early stage. Cellulose has only gained prominence as a nanostructured material over the past few years. This market is still under development and has a number of shortcomings, especially for high-volume applications, such as those in the building sector. For this reason, no industrial commercial applications have been implemented to date. However, this situation is expected to change in a few years; for instance, the CNF market is growing in Japan with paper manufacturers, such as Nippon Paper and Oji Holdings, and Japanese chemical manufacturers establishing large nanofiber production facilities. Facilities have also been recently established in Europe, Canada, and the United States; however, Japan is by far the largest current market for cellulose nanofiber producers, product developers, and products.

An impediment to commercial progress with CNF is cost-competitiveness with traditional technology and the availability of volumes relevant for large-scale industrial use, such as in building products. The high energy consumption needed to produce CNF has so far prevented it from competing with other mass products, such as cellulose or synthetic polymers. However, these challenges are being overcome and CNF is expected to have a substantial impact in high-volume applications. The main applications that are currently targeted by most producers include reinforcing agents in paper, cement, natural rubber, biocomposites, and plastic films for packaging. The recent developments in energy-efficient and up-scalable production methods developed by a number of companies have significantly reduced the cost of production. Currently, most CNF producers sell cellulose nanofibers for less than $100/kg, being $9/kg the minimum market price so far. This does not make CNF competitive against current competitive materials in many industries; however, the effort to develop low-cost production methods or target higher-value applications could change this situation in a few years, especially if the price is reduced below $1.5/Kg [142].

In this respect, most of the major paper producers view the commoditization of NC as achievable. Production to date is, in the majority of companies, on a pilot scale and pre-commercial. Many paper producers are planning to construct large commercial production facilities in the near future. This could lead to a significant market change both in terms of availability and price. The end goal of most major producers is bulk supply rather than niche or low-volume applications; as a consequence, the price will continue to drop over the next few years and open up possibilities in other sectors, such as those that deal with cement and fiber-reinforced materials.

## 5. Future Research Requirements

Dispersion is a requirement for success in using NC, since most nanocrystals and nanofibrils tend to self-agglomerate via hydrogen bonding and van der Waals forces. NC must be dispersed in the water phase before mixing it with the cement, but this will affect the rheology of the water phase and can cause difficulties when mixing it with the solid phase. If NC is not properly dispersed, the aggregates can induce the formation of large pores in the composite, and they act as stress concentration points under loading conditions [76,84,85]. Dispersion is even more difficult in the case of BC, with a high energy requirement to disperse the gel, and CNCs, which have a high tendency to agglomerate themselves [143]. Decreasing the NC concentration could be an effective way to improve the NC dispersion while decreasing the energy requirements; however, it is not feasible when the ratio water/cement is low. This limits the amount of NC that can be used. Moreover, the analyzed studies show that the reinforcing effect of NCs is related to their aspect ratio, which provides a complete dispersion of the NC. However, mechanical entanglement and NC network formation increase with the aspect ratio, which increases the difficulty of its dispersion.

The use of ultrasonication, high shearing forces, and dispersing agents are the most common ways to improve NC dispersion in the slurry [84,85,86]. However, there are few studies on the effect of dispersion of CNF on cementitious composites and the question of how to optimize their dispersion in the highly viscous water–cement slurry. Another strategy to improve dispersion is the functionalization of the NC surface to create disruption electrostatic forces and to increase the interaction between the NC and the matrix. This has been proven by Anju, Ramamurthy, and Dhamodharan [89] to be efficient when MCCs were used to reinforce cement mortar.

Despite the requirement of fast drainage in a Hatscheck machine, there is a lack of knowledge about the effect of the use of NC on the drainage of the C-FCCs slurry. It is known that the use of NCs in papermaking can notably decrease the drainage rate and this is one of the drawbacks for their application in papermaking [64]. As has been recently proven in papermaking, NC retention and drainage can be decoupled by changing the retention aids [144,145]. This is an important issue that could be controlled online by means of Focus Beam Reflectance Measurements [146,147]. Furthermore, the type of flocculant is also relevant to flocculant resistance and reflocculation capacity [148,149]. However, no studies exist on how the use of NC affects the retention and drainage in the Hatscheck process. In contrast to papermaking, the C-FCC suspension consists mainly of small inorganic particles and a few fibers. This could reduce the effect of adding NC to the drainage process and quite likely increase its effect on retention due to the high increase in the surface area available to interact with the mineral particles. However, this is only a prediction based on previous studies on the effect of fiber morphology on drainage carried out by Tonoli et al. [150], who observed that pulp refining decreases the drainage rate of the fiber cement suspensions; however, it notably improved the solids retention.

Finally, one of the most common worries related to the use of NC in low-value-added products is the high cost. Although the price of NC is controversial and the given values are very different from one author to another depending on the kind of NC production, the NC properties, and the sources taken into account for the economic analysis, there is the common perception that it is too expensive for the manufacture of low-value-added products. There is increasing interest in three strategies to reduce the costs of using NC in these kind of processes: (1) reducing the cost by simplifying the pretreatment in the production of CNF by using new methods for hydrolysis, separation in the production of CNC, or plant wastes and novel bioreactor designs in the production of BC [14]; (2) in situ production by means of simple treatments that allow us to use the facilities and equipment already existing in the mill when possible or at least to avoid the costs of transporting/removing the water contained in the NC suspension, which can be over 99 wt.%; and (3) fitting the properties of NC for the specific requirements, which includes their functionalization to enhance certain properties or reduce dispersion or fibrillation costs.

## 6. Conclusions

Although the performance of C-FCCs has been improved in the last few decades, new strategies are required to further improve the durability and the mechanical properties of these composites, while developing ecofriendly technologies. In this context, bio-based nano-structurated cellulose materials, such as NC, can open up new solutions to overcome these limits. The addition of NC as an additive in fiber cement production has been demonstrated to be efficient in improving mechanical properties, such as internal bonding strength, MOE, and MOR, modifying the slurry’s rheology, reducing the porosity, and driving interactions with other components of the slurry. However, many variables must be considered when NCs are used to reinforce fiber–cement composites. The type of cellulose fiber, the surface chemistry and morphology of NC, the dosage of NC, the way that NCs are incorporated into the composite, the curing process, and the morphology and nature of the components of the matrix, such as sand, aggregates, or silica, all have an important impact on the results. Only a small part of the huge variety of possible combinations of these factors has been studied, and it has been demonstrated that NCs have great potential to improve the mechanical properties of C-FCCs, mortars, and other cementitious composites. A greater research effort on underexplored combinations could reveal a great diversity of possibilities for sustainable natural-based fiber-reinforced cementitious composites.

Future studies are required in order to evaluate the effects of NC as a surface treatment agent, an anionic flocculant, or an additive for wastewater treatment in the fiber cement industry.

Finally, the main drawbacks of NCs are their cost, when compared to other traditional strategies, and their availability in large quantities, when they are to be used in high-volume applications. However, in the short term, these perspectives allow us to consider as likely the production of NC in large quantities and thus a drop in its cost, which would open up possibilities in the building sector.

## Figures and Tables

**Table 1:** Effect of nanocelluloses (NCs) in cement and fiber-cement composites.

NC Type	Source NC	NC Dose (wt.%)	Cementitious Material	Effect on Mechanical Properties	Other Effects	Ref.
**Cellulose nanofibers (CNFs)**						
High intensity refining process in a Valley Beater Refining time: 6 h d = 25–250 nm	Sisal (*Agave sisalana*)	3.3	**Cement mortar** - Type I cement - Sand - Ratio (C:S:W) = 1:1:0.67	ΔMOE (%) = 70.83 * ΔMOR (%) = 35.92 * ΔFracture Energy (%) = −52.96 *		[71]
		4	**Cement mortar** - Type I cement - Silica Fume - Sand - Ratio (C:Si:S) = 0.9:0.1:1 - Ratio (W:C) = 0.6	ΔMOE (%) = 30.77 * ΔMOR (%) ≈ 5 * ΔFracture Energy (J) ≈ −83.33 *		[73]
		2	**Fiber cement** - Type I cement - Silica Fume - Cellulose fibers (2 wt.%) - Sand - Ratio (C:Si:S) = 0.9:0.1:1 - Ratio (W:C) = 0.7	ΔMOE (%) = 50 * ΔMOR (%) ≈ −5 * ΔFracture Energy (%) ≈ −46.67 *		[73]
		8	**Cement mortar** - Type I cement - Silica Fume - Sand - Ratio (C:Si:S) = 0.9:0.1:1 - Ratio (W:C) = 0.56	ΔMOE (%) = 55.55 * ΔMOR (%) = 37.07 * ΔFracture Energy (%) = −90.55 *		[70]
		2–6	**Fiber cement** - Type I cement - Silica Fume - Cellulose fibers (2–6 wt.%) - Sand - Ratio (C:Si:S) = 0.9:0.1:1 - Ratio (W:C) = 0.56–0.69	ΔMOE (%) = 27.78–113.89 * ΔMOR (%) = 3.45–23.27 * ΔFracture Energy (%) = −51.32–(−83.88) *		[70]
		3.4	**Cement mortar** - Type I cement - Silica fume - Sand coarse - Ratio (C:Si:S) = 0.7:0.3:1 - Ratio (W:C) = 0.84	ΔMOE (%) = 10.17 * ΔMOR (%) = 1.55 * ΔFracture Energy (%) = −81.90 *		[74]
		3.3	**Cement mortar** - Type I cement - Silica fume - Sand fine - Ratio (C:Si:S) = 0.7:0.3:1 - Ratio (W:C) = 0.89	ΔMOE (%) = 60.71 * ΔMOR (%) = 6.06 * ΔFracture Energy (%) = 6.45 *		[74]
Bleaching (NaClO) Deproteinization (NaOH) and removing of oil and pigments (CHCl_3_) Demineralization (HCl) d = 37–55 nm	Waste algae (*Cladophora* sp.)	0.1–1.0	**Cement mortar** - Portland cement - Sand - Ratio (C:S:W) = 1:4:1	ΔMOE (%) = 3.62–169.68 (adding 0.1 and 1.00 wt.% CNF, respectively)		[75]
Commercial supplied by Sigma-Aldrich, CAS: 9004-34-6 d < 35 nm	Cotton	0.1–1.0	**Cement mortar** - Portland cement - Sand - Ratio (C:S:W) = 1:4:1	ΔMOE (%) = −37.10–(−36.20) (adding 0.1 and 1.00 wt.% CNF, respectively)		[75]
TEMPO oxidation and fibrillation L = 0.6–1.7 µm d = 20–100 nm COOH = 1850 µmol/g	Bleached hardwood pulp	0–0.4	**Cement mortar** - Type I cement - Ratio (W:C) = 0.5	ΔCompression strength (%) = 20 ΔFlexural strength (%) = 15 Both with the optimal dose of CNF (0.15 wt.%)	The porosity notably decreased with the increasing dose of CNF	[74]
TEMPO oxidation and fibrillation at 600 bar at 1.5% to obtain a gel (5–6 cycles) L = 1–2 µm d = 5–10 nm COOH = 500 µmol/g,	Bleached Eucalyptus Kraft	0–0.5	**Cement mortar** - Type I cement 32.5 N EN197-1:2000 - Ratio (W:C) = 0.26	ΔCompression strength (%) = 43	ΔHardening (%) = 66 ΔConductivity (%) = 36 ΔPorosity (%) = −36	[76]
- TEMPO oxidation and grinding at 1 wt.% - COOH = 1130 µmol/g,	Bleached Eucalyptus chemithermomechanical pulp	0–1.2	**Cement mortar** - Portland Type II cement C3A < 8% - Ratio (W:C) = 0.485		ΔYield stress (%) = 94 (0.2 wt.% CNF) ΔPlastic viscosity (%) = 25 (0.2 wt.% CNF) Reduced bleeding and leaching ΔCrack area (%) = 27 (0.8 wt.% of CNF)	[77]
- Cellulose filaments (CFs): Commercial supplied by Kruger Biomaterials Mechanical fibrillation L = 100–2000 µm d = 20–200 nm	Wood	0–0.2	**Cement mortar** - General used cement (GU) - Fly ash (FA) - Ratio (GU:FA:W) = 1:0.33:0.5	ΔCompression strength (%) = −20 ΔFlexural strength (%) = 38 (with 0.2 wt.% CF)	ΔSlump (%) = −61 (with 0.2 wt.% CF) ΔPlastic viscosity (%) = 41 ΔAutogeneous shrinkage (%) = −43	[78]
		0–0.2	**Self-consolidating concrete** - General used cement (GU) - Sand (S) - Coarse aggregate (CA) - Superplasticizer (SP) - Ratio (GU:S:CA:SP:W) = 1:0.33:0.5:2:2.15	ΔCompression strength = 16% ΔFlexural strength = 28% ΔEnergy absorption = 96% with 0.2 wt.% CF	ΔSlump (%) = −50 (with 0.2 wt.% CF) ΔPlastic viscosity (%) = 113 ΔYield stress (%) = 794	[78]
Disc grinding method Grinding cycles: 15 L = 1–2.5 µm d = 20–200 nm	Bleached softwood pulp	0–0.4	**Limestone based cement** - Ratio (W:C) = 0.5 - Superplasticizer (1.6 wt.% on cement)	ΔFlexural strength = 106% ΔEnergy absorption = 186% with 0.1 wt.% of CNF	ΔHardening (%) = 10	[79]
Super Masscolloider method Grinding cycles: 35 d (55%) = 40 nm, Mean d = 50 nm	Bleached Eucalyptus Kraft	0, 0.5, 1	**Extruded cement** Cement plus limestone	ΔMOE and ΔMOR insignificant changes Δabsorbed Energy (%) = −72 (with 1wt.% CNF)	ΔPorosity (%) = 95 (with 1 wt.% CNF) ΔWater absorption ability (%) = 80 (with 1 wt.% CNF)	[80]
Grinding method Grinding cycles: 10 L > 1 µm mean d = 16.2 nm	Unbleached bamboo organosolv	1	**Fiber cement** - Type I cement - Limestone filler - Unbleached bamboo organosolv pulp (9 wt.%) - Ratio (C:Li) = 3:1 - Ratio (W:C) = 0.57	ΔMOE (%) = 5.20 * ΔMOR (%) = 34.46 * ΔFracture Energy (%) = 6.86 * ΔFracture toughness (%) = 12.74 *	Significant effects were not observed on density and porosity	[81]
Chemical and mechanical pretreatment Homogenization	Pine Kraft	0.14, 0.27, 0.41	**Cement mortar** - Portland-limestone cement (CEM II/A-LL 42.5R) - Sand - Superplasticizer - Anti-washout admixture - Ratio (W:C) = 0.6 - Ratio (C:S:W) = 1:1.07:0.6	ΔMOR (%) = 4.93–18.64 ΔCompressive Strength (%) = 3.96–4.56	ΔMini-slump flow (%) = −14.54–(−24.73) ΔYield stress (%) = 100–142.10 ΔPlastic viscosity (%) = −40–200	[82]
Only chemical treatment L = 1.1. mm Mean d = 45 µm L/d = 24.4 Density = 30 kg/m^3^	Recycled cartonboard	0.045	**Cement mortar** - CEM I CALCIA cement 52.5 N - Sand (0.125–4 mm) - Filler (F): calcium carbonate - Superplasticizer: modified polycarboxylate -Ratio (W:C) = 0.48 -Ratio (C:S:F) = 1:2.6:0.37	ΔMOR (%) = 4.35 ΔCompressive Strength (%) = 10	ΔWater porosity (%) = 12.5	[83]
		5.7	**Cement concrete** - CEM I CALCIA cement 52.5 N 2 - Sand (0.125–4 mm) plus Gravel (G) (4–16 mm) - Filler (F): calcium carbonate - Superplasticizer: modified polycarboxylate -Ratio (W:C) = 0.48 -Ratio (C:S+G:F) = 1:4.65:0.37	ΔCompressive Strength (%) = 25	ΔMercury intrusion porosimetry (%) = −7.25 ΔPermeability (%) = −25	[83]
Microcellulose Sigmacell 101 Nanocellulose in gel at 3%	unknown	1, 3, 5	**Reactive powder concrete** - Sand - Silica flour - Silica fume - Superplasticizer 1.6% on cement -Ratio (C:S:Sflour:Sfume:) = 1:0.98:0.28:0.39 -Ratio (W:C) = 0.22–0.35	ΔMOE and ΔMOR insignificant changes with 1% of micro or nanocellulose ΔFracture Energy (%) = 24 * with 2 wt.% of mixture micro-nanocellulose ΔFracture Energy (%) = 50 * with 3 wt.% of microcellulose		[72]
**Cellulose micro and nanocrystals (MCCs and CNCs)**						
- CNC Produced via sulfuric acid hydrolysis 0.814 wt.% surface sulfate content Freeze-dried powder	Eucalyptus	0–0.77	**Cement paste** - Type V cement - Ratio (W:C) = 0.35	ΔMOR (%) = 20 with 0.10 wt.% of CNC (max improvement)	ΔYield stress (%) = from −67.2 (with 0.02% of CNC) to 1137 (with 1.5 wt.% of CNC) ΔCumulative heat (%) = 16 with 0.77 wt.% of CNC (at an age of 200 h) ΔPorosity (%) = −16% ΔDegree of hydration (%) = 20 with 0.77 wt.% of CNC	[84]
CNC Produced via sulfuric acid hydrolysis 0.814 wt.% surface sulfate content Dispersed in water by sonication	Eucalyptus	0–0.77	**Cement paste** - Type V cement - Ratio (W:C) = 0.35	ΔMOR (%) = 23 with 0.10 wt.% of CNC, but 30 with 0.5 wt.% of CNC	ΔPorosity (%) = −16%	[85,86]
Commercial MCC, Sigma Aldrich	Cotton	0, 3	**Cement mortar** - Portland cement - Ratio (W:C) = 0.45	ΔCompressive strength (%) = −12 ΔFlexural strength (%) = −25	ΔCritical yield stress (%) = 155	[87]
MCC (Avicel^®^ PH101) plus Carbon nanotubes (CNTs) L = 2–260 µm mean d = 49.1 µm (MCC) L = 10–30 µm d (inner) = 2–5 nm; d (outer) < 8 nm (CNTs)	Cotton linters	0.2 (+ 0.1 wt.% CNTs)	**Cement mortar** -Portland cement (CEM I 42.5R) -Sand (NP-EN 196-1) - 1.5 wt.% Plutonic F-127 surfactant to dispersed MCC plus CNTs in water - 0.75 wt.% defoamer (tri-butyl phosphate (TBP)) to suppress the foam formation -Ratio (W:C) = 0.5 -Ratio (C:S:W) = 1:3:0.5	ΔMOR (%) = 2.9 ΔMOE (%) = 24.1 ΔFracture Energy (%) = 16.1 ΔCompressive Strength (%) = 16.98	ΔDry bulk density (%) = 8.24 ΔPore diameter = −40.51 ΔPorosity = 32.38	[88]
		0.5 (+ 0.3 wt.% CNTs)	**Cement mortar** -Portland cement (CEM I 42.5R) -Sand (NP-EN 196-1) - 1.0 wt.% CTAB surfactant to dispersed MCC plus CNTs in water - 1.0 wt.% defoamer (TBP) to suppress the foam formation -Ratio (W:C) = 0.5 -Ratio (C:S:W) = 1:3:0.5	ΔMOR (%) = 12.3 ΔMOE (%) = 12.7 ΔFracture Energy (%) = 85.2 ΔCompressive Strength (%) = 16.26	ΔDry bulk density (%) = 4.49 ΔPore diameter = −36.08 ΔPorosity = 22.86	[88]
MCC Sulphuric acid solution L = 75–400 µm L > 150 µm, for about 40% of MCC; d = 10–30 µm Maximum weight loss temperature (°C) = 300	Cotton linters	2.5	**Cement mortar** - Portland cement - Sand - Ratio (W:C) = 0.45 - Ratio (C:S) = 1:3	ΔMOR (%) = 50 ΔCompressive Strength (%) = −21.45		[89]
		2.5	**Cement mortar** - Portland cement - Sand -Superplasticizer: polycarboxylate ether (1 wt.% of cement) - Ratio (W:C) = 0.4 - Ratio (C:S:W) = 1:2.5:0.4	ΔMOR (%) = 16 ΔCompressive Strength (%) = −9.47		[89]
Tetraethyl orthosilicate (TEOS) surface-modified MCC Sulphuric acid solution TEOS as silane agent L = 75–400 µm L > 150 µm, for about 40% of MCC; d = 10–30 µm Maximum weight loss temperature (°C) = 360	Cotton linters	2.5	**Cement mortar** - Portland cement - Sand - Ratio (W:C) = 0.45 - Ratio (C:S) = 1:3	ΔMOR (%) = 94 ΔCompressive Strength (%) = 45		[89]
			**Cement mortar** - Portland cement - Sand -Superplasticizer: polycarboxylate ether (1 wt.% of cement) -Ratio (W:C) = 0.4 -Ratio (C:S) = 1:3	ΔMOR (%) = 59 ΔCompressive Strength (%) = 57		[89]
Commercial MCC (Sigma Aldrich) Bulk density = 0.459 g/mL	-	3	**Cement mortar** - Portland Low Alkali CP40 cement - Sand -Ratio (W:C) = 0.45 -Ratio (C:S) = 1:2.7	ΔMOR (%) = −26.31 ΔCompressive Strength (%) = −13.46	ΔDensity (%) = −2.16 ΔDiameter (mini-slump test)(%) = −17.65 ΔYield stress (mini-slump test) (%) = 160 ΔGlobal heat transference coefficient (%) = −24 ΔTemperature adiabatic max (%) = −5.28	[90]
**Bacterial cellulose (BC)**						
		0.02	**Cement mortar**	ΔMOR (%) = 20 ** ΔCompressive strength (%) = 8 **	Accelerated production of calcium silicate hydrate (CSH)	[91]
Zetasizer (75 nm) crystallinity (DXR) = 65% BC was used for coating bagasse fibers before mixing	*Gluconacetobacter xylinus* cultured in Hestrin–Schramm medium	0, 3	**Fiber cement** - Portland Type II - 5% CaCl_2_ - Unbleached bagasse fibers (6 wt.%) -Polycarboxilated superplasticizer	ΔMOE (%) = 38 ** ΔMOR (%) = 68 ** ΔInternal Bonding strength (%) = 50 ** ΔFracture toughness (%) = 70 **	Decreased fiber mineralization Increased durability Decreased porosity Great increase in the surface basicity	[92,93]
Zetasizer (75 nm) crystallinity (DXR) = 65% Freeze-dried powder	*Gluconacetobacter xylinus* cultured in Hestrin–Schramm medium	0, 3	**Fiber cement** - Portland Type II - 5% CaCl_2_ - Unbleached bagasse fibers (6 wt.%) -Polycarboxilated superplasticizer	ΔMOE (%) = 11 ** ΔMOR (%) = 47 ** ΔInternal Bonding strength (%) = 10 ** ΔFracture toughness (%) = 60 **	Decreased fiber mineralization, surface porosity, and surface roughness	[92,93]
Zetasizer (75 nm) crystallinity (DXR) = 65% BC dispersed in water forming a gel	*Gluconacetobacter xylinus* cultured in Hestrin–Schramm medium	0, 3	**Fiber cement** - Portland Type II - 5% CaCl_2_ - Unbleached bagasse fibers (6 wt.%) -Polycarboxilated superplasticizer	ΔMOE (%) = 33 ** ΔMOR (%) = 58 ** ΔInternal Bonding strength (%) = 30 ** ΔFracture toughness (%) = 40 **	Decreased fiber mineralization Increased durability Decreased porosity, surface basicity, and roughness Increased the organic compounds on the surface	[92,93]

(*) with respect to the properties reached with the same dosage of cellulose fibers; (**) with respect to the properties reached without NC. MOE, modulus of elasticity; MOR, modulus of rupture.

**Table 2:** Comparison of refining versus using NC in fiber-cement composites.

	Refining	NC
Production requirements	High energy demands	High energy demand and/or chemical reactives
Type and properties of cellulose fibers produced	Cellulose fibers with internal and external fibrillation Production of fines	Nano- or microcellulose fibers No fines production
	Medium specific surface area	Very high specific surface area
	Macroscale dimensions	Nanoscale dimensions
	Length reduction (cutting)	Length and diameter reduction
	Formation of hydrogen bridges	High tendency to form hydrogen bridges
	Increases swelling ability	Very high swelling ability, gel formation
Chemical modification	Easy (after refining)	Even during production, many different possibilities for chemical modification
Cracks prevention	Macrocracks	Microcracks
Interactions	Increasing the capacity of the cellulose fibers to bond with cement matrix	Highly reactive with the cellulose fibers and the cement matrix, coating the cellulose fibers
Mechanical properties	Improves mechanical properties and network strength	Highly improved mechanical properties in combination with the cellulose fibers
Durability	Increases durability, reducing strength losses by increasing interaction with the matrix	Increases durability: preventing lumen mineralization, increasing interaction with the matrix, decreasing porosity
Drainage	Decrease in the drainage rate	It likely decreases the drainage rate; however, there are no studies on that in C-FCCs
